# Aggregation-induced emission: challenges and opportunities

**DOI:** 10.1093/nsr/nwaa222

**Published:** 2020-08-31

**Authors:** Wenbo Wu, Bin Liu

**Affiliations:** Institute of Molecular Aggregation Science, Tianjin University, China; Department of Chemical and Biomolecular Engineering, National University of Singapore, Singapore; Joint School of National University of Singapore and Tianjin University, International Campus of Tianjin University, China

**Figure 1.**
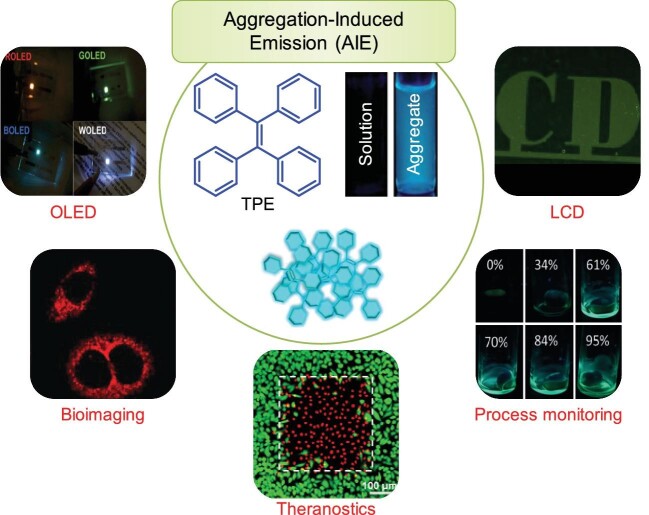
AIE phenomenon and application. Adapted with permission from Ref. [1,3,4]. Copyright 2013, American Chemical Society; Copyright 2020, Wiley-VCH; Copyright 2018, Wiley-VCH.

Luminescent materials are everywhere in our daily life. However, most are not performing at their best performance because of the thorny aggregation-caused fluorescence quenching (ACQ) effect in molecular aggregates. Opposite to ACQ, aggregation-induced emission (AIE) describes the photophysical phenomenon in which non-luminescent molecules in solutions are induced to show bright emission upon aggregate formation, as a result of restriction of intramolecular motions (RIM) caused by intermolecular steric interaction (e.g. tetraphenylethene (TPE) in Fig. 1) [1].

Compared to traditional ACQ fluorophores, the greatest advantage of AIE luminogens (AIEgens) is the highly efficient radiative transition in aggregate state for practical applications. Over the past 20 years, many researchers have focused on developing AIEgens, which have demonstrated great potential in different areas from organic electronics to biomedical research and physical process monitoring (Fig. 1). From traditional light-emitting materials to aggregation-induced delayed fluorescent (AIDF) molecules, AIEgen based organic light-emitting diodes (OLEDs) show bright luminescence, high current efficiency and excellent external quantum efficiency with very low efficiency roll off, showing great potential for commercialization [2]. Of note, chiral AIDF molecule-based LEDs can directly show circularly polarized electroluminescence with high efficiency for three-dimensional stereoscopic display [2].

AIE liquid crystals with bright emission could be used to fabricate liquid crystal displays without the need for backlight to reduce energy consumption [2].

Similarly, the potential of AIE has been greatly exploited to develop AIE light-up molecular probes, AIE dot probes and AIE theranostic probes for continuous monitoring of biological processes, disease diagnosis, cell tracking, vascular imaging, image-guided surgery and therapy [3]. Recently, the light-up response of AIEgens was also used successfully for physical process monitoring

(e.g. glass transition, polymerization, micelle formation, gelation, self-assembly, etc.) with RIM caused by microenvironment changes [4], an interesting research direction worthy of further investigation.

Despite the notable success in fundamental study and applications of AIEgens in the past two decades, it should be noted that there are challenges to use of AIEgens. We start with emission, the most distinctive feature of AIEgens. When AIEgens are randomly packed, broad emission is often observed because the torsional structure and changeable conformation can endow AIEgens with abundant energy levels, affecting the color purity in OLEDs. The broad emission can also cause cross-talking in multiplex sensing and imaging. A recent study revealed that adopting fluorophores or quantum dots with very sharp emission bandwidth to form donor-acceptor pairs with AIEgens can offer narrow emission output through Förster resonance energy transfer (FRET) [5]. In addition, integration of AIEgens with photonic crystals can also narrow the emission spectrum as the photonic band gap structure can make light in certain wavelengths completely unable to propagate [5].

The short absorption wavelength of most reported AIEgens is often regarded as a disadvantage. This is largely because of their twisted molecular structures, which may not affect their performance in electronics, but can significantly reduce the penetration depth in biomedical research, especially for *in vivo* applications. Red-shifting the absorption by precisely adjusting the molecular structure is the most straightforward solution to the problem, and one successful example was to integrate rotor structure with twisted intramolecular charge so that AIEgens with more than 700 nm absorption peak were designed for *in vivo* near-infrared II bioimaging [6]. The development of multiphoton absorbing AIEgens and the integration between AIEgens and upconversion nanomaterials could also help bring the excitation wavelength to near-infrared range [5]. The excitation wavelength dependence is eliminated when chemiluminescence is triggered to excite the AIEgens [3]. In addition, the twisted molecular structures may also endow AIEgens with relatively low molar extinction coefficients, which impact their effectiveness in light absorption. Clearly, planarization of the conjugated system and increasing the conjugation length are the two most effective strategies at present [7]. Polymerization of AIEgens into conjugated polymers is another strategy to enhance the molar extinction coefficients [8].

**Figure 2.**
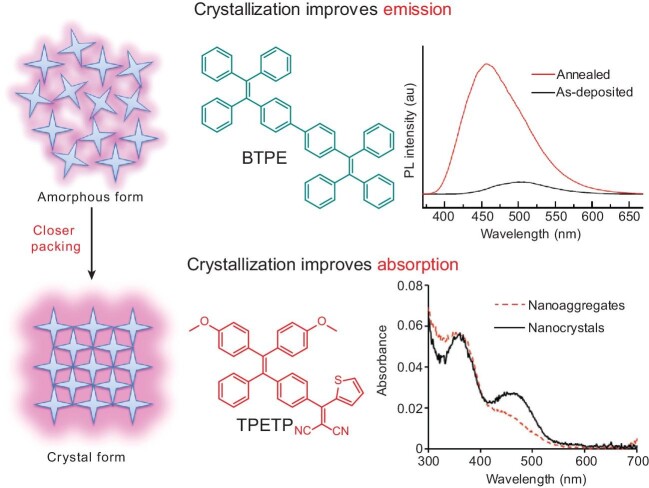
Improvement in emission and absorption of AIEgens by crystallization. Adapted with permission from Ref. [9,10]. Copyright 2012, Elsevier; Copyright 2018, Wiley-VCH.

Recent work also revealed that the molecular motions of AIEgens are still inevitable even in aggregate states. Through introducing spacers, molecular motions in the aggregate state have been used to design effective AIE photothermal agents for cancer therapy [3]. This new observation further indicates that inhibition of molecular motions in aggregate states should be taken into consideration for optimized AIE emission. Crystallization is the general and simple method to realize closer and ordered molecular packing (Fig. 2) [8]. Over 10-fold improvement in brightness of 4,4'-bis(1,2,2-triphenylvinyl)-1,1'-biphenyl (BTPE) films was achieved by simply annealing, and the formation of nano-wire shaped crystals led to 31.5% improvement in OLED efficiency (Fig. 2) [9]. In addition, crystallization could also help to enhance the molar extinction coefficients with red-shifted absorption maxima for some AIEgens. For example, the absorption peak of 2-((4-(2,2-bis(4-methoxyphenyl)-1-phenylvinyl)phenyl)(thiophen-2-yl)methylene)malononitrile (TPETP) showed 50% enhancement in absorbance with 60 nm red-shift in absorption maximum after nanocrystallization (Fig. 2) [10].

Exploration of AIE applications in healthcare, energy and environmental fields has led to the flourish of many research frontiers. For biomedical research, aggregation-enhanced reactive oxygen species generation of AIE photosensitizers is a very promising strategy to improve photodynamic efficiency in tumor therapy, and the molecular motions in the aggregate state can be well-controlled through molecular design to build photothermal systems with high conversion efficiency for photoacoustic imaging and photothermal therapy. In these applications, red-shifting the absorption of AIEgens to near-infrared range to match the parameters of commercial lasers will shed light on potential clinical translation. Considering the metabolism issue, exploration of clusteroluminescence from non-conjugated AIEgens or even natural products represents another promising pathway to translation. An important parameter not given the same consideration is their absorption. More effective absorption means better light harvesting, and therefore less material may be needed for the same application, which could help improve biocompatibility. On the other hand, mechanoluminescence and organic room-temperature phosphorescence are two new branches closely associated with the concept of AIE, which show great potential in detection, anti-counterfeiting, information encryption, afterglow imaging, etc. These studies require us to be equipped with strong capability in designing molecules that can easily undergo crystallization and may even form different types of crystals. When proper strategies are used to control or narrow down the emission spectra of mechanoluminescence and organic room-temperature phosphorescence, the anti-counterfeiting and information encryption properties can be further improved as a result of minimized overlap among the output signals. Last but not least, chiral AIEgen-based circular polarization LEDs can significantly reduce brightness loss when passing through polarizers, which has great potential to enhance efficiency in 3D display and optical communication.

The enrichment of mechanism studies of AIEgens, including anti-Kasha decay, restriction of flip-flop motion, through-space interaction, etc., will continue to play an important role in shaping the AIE field. This will inspire more design principles to be developed for AIEgens and empower them with further improved functions and properties to solve practical problems. It is also important to note that the emergence of new and complementary theories to explain the AIE mechanism is a natural phenomenon. The in-deep mechanism research, coupled with modelling, big data and artificial intelligence will bring AIE research to the next level of excellence in materials innovation.

The past 20 years have witnessed rapid development of the AIE field, from both fundamental science and practical applications. In this perspective, we focused on several challenges of AIEgens and discussed potential solutions to address these issues. It is important to note that the future directions mentioned are evolving with time, and integration between AIE and any new emerging research field can create new multi-disciplinary directions at any time. With artificial intelligence powered design and synthesis of AIEgens, the field is expected to have exponential growth in the next decade. By addressing the discussed challenges, AIEgens will become more powerful in addressing national challenges in energy, environment and social security, making an even broader impact on our everyday life.
